# *Pinus* Susceptibility to Pitch Canker Triggers Specific Physiological Responses in Symptomatic Plants: An Integrated Approach

**DOI:** 10.3389/fpls.2019.00509

**Published:** 2019-04-24

**Authors:** Joana Amaral, Barbara Correia, Carla António, Ana Margarida Rodrigues, Aurelio Gómez-Cadenas, Luis Valledor, Robert D. Hancock, Artur Alves, Glória Pinto

**Affiliations:** ^1^Department of Biology, Centre for Environmental and Marine Studies, University of Aveiro, Aveiro, Portugal; ^2^Plant Metabolomics Laboratory, Instituto de Tecnologia Química e Biológica António Xavier, Universidade Nova de Lisboa, Oeiras, Portugal; ^3^Departament de Ciències Agràries i del Medi Natural, Universitat Jaume I, Castelló de la Plana, Spain; ^4^Plant Physiology, Department of Organisms and Systems Biology, University of Oviedo, Oviedo, Spain; ^5^Cell and Molecular Sciences, James Hutton Institute, Dundee, United Kingdom

**Keywords:** biotic stress response, disease differential susceptibility, forest tree disease, gene expression, plant hormones, plant physiology, plant primary metabolism

## Abstract

*Fusarium circinatum*, the causal agent of pine pitch canker (PPC), is an emergent and still understudied risk that threatens *Pinus* forests worldwide, with potential production and sustainability losses. In order to explore the response of pine species with distinct levels of susceptibility to PPC, we investigated changes in physiology, hormones, specific gene transcripts, and primary metabolism occurring in symptomatic *Pinus pinea*, *Pinus pinaster*, and *Pinus radiata* upon inoculation with *F. circinatum*. *Pinus radiata* and *P. pinaster* exhibiting high and intermediate susceptibility to PPC, respectively, suffered changes in plant water status and photosynthetic impairment. This was associated with sink metabolism induction, a general accumulation of amino acids and overexpression of pathogenesis-related genes. On the other hand, *P. pinea* exhibited the greatest resistance to PPC and stomatal opening, transpiration increase, and glycerol accumulation were observed in inoculated plants. A stronger induction of pyruvate decarboxylase transcripts and differential hormones regulation were also found for inoculated *P. pinea* in comparison with the susceptible *Pinus* species studied. The specific physiological changes reported herein are the first steps to understand the complex *Pinus–Fusarium* interaction and create tools for the selection of resistant genotypes thus contributing to disease mitigation.

## Introduction

Pine pitch canker (PPC), caused by *Fusarium circinatum*, affects *Pinus* species and *Pseudotsuga menziesii* worldwide (Wingfield et al., 2008; European Food Safety Authority [EFSA], 2010). In nurseries its symptoms include damping-off and wilting of seedlings and, on mature trees, branch die-back, stem cankers, pitch formation and mortality (Wingfield et al., 2008). More than 10 million ha are potentially threatened by PPC in Europe (European Food Safety Authority [EFSA], 2010), where it is recommended as a quarantine pathogen (Decision 2007/433/EC of 18 June 2007). Moreover, climate change may shift *F. circinatum* distribution toward Europe (Watt et al., 2011), endangering *Pinaceae* presently in pathogen-free areas such as *Picea abies* (Martín-García et al., 2017). Given the significance of conifer forests worldwide, the threat posed by *F. circinatum* should be urgently considered.

Although methods such as *Trichoderma* and phosphite application (e.g., Cerqueira et al., 2017; Martín-García et al., 2017) have been proposed to mitigate the disease, no effective solutions have yet been found. Studies focussing on the characterisation of *F. circinatum* populations, isolate pathogenicity (e.g., Iturritxa et al., 2011; Berbegal et al., 2013), and host susceptibility trials are also abundant. It is well-known that *Pinus* susceptibility to *F. circinatum* is species-dependent. Several independent experiments worldwide indicate that *Pinus radiata*, *Pinus patula*, and *Pinus elliottii* are highly susceptible to PPC, while *Pinus tecunumanii*, *Pinus oocarpa*, *Pinus canariensis*, *Pinus pinea*, and *Pinus thunbergii* are highly resistant (reviewed by Martín-García et al., 2019). The Mediterranean *Pinus pinaster* presents and intermediate response between *P. radiata* and *P. pinea* (Bragança et al., 2009; Iturritxa et al., 2013; Martínez-Álvarez et al., 2014). Most of these studies follow symptoms development, mortality rates, or lesion length during disease progression, but the mechanisms behind these differential responses remain unknown.

Few reports on *Pinus* response mechanisms against *F. circinatum* infection are available. Cerqueira et al. (2017) unveiled physiological and hormonal changes occurring in *P. radiata*. Other studies explored gene expression regulation during PPC progression using either high-throughput mRNA sequencing (Carrasco et al., 2017) or targeted approaches with special focus on secondary metabolism and phenylpropanoid pathway induction (Davis et al., 2002; Morse et al., 2004; Fitza et al., 2011, 2013; Donoso et al., 2015). Surprisingly, although the importance of primary metabolism regulation to fuel plant defence mechanisms against pathogen attack is well-documented (Berger et al., 2007; Bolton, 2009; Rojas et al., 2014), few studies have addressed this area in the *Pinus–F. circinatum* interaction. The only study of which we are aware examined carbohydrate content changes in *P. pinaster* upon *F. circinatum* inoculation (Vivas et al., 2014).

In general, pathogen attack induces changes in energy-producing primary metabolism, plant defence, and hormone signalling either as a direct result of pathogenesis or plant defence mechanisms. Photosynthesis limitation, often associated with *Ribulose 1,5-biphosphate carboxylase/oxygenase small subunit (RuBisCO)* down-regulation, usually occurs in association with the induction of defence-related pathways (Bilgin et al., 2010; Rojas et al., 2014). In a scenario of increased energy demands, photosynthesis repression may lead to source-to-sink tissue transformation (Bolton, 2009). Cell wall invertase (cwInv) activity, cleaving sucrose into fructose (Fru) and hexose, is crucial for this carbohydrate metabolic shift (Proels and Hückelhoven, 2014). Sugars have been shown to be important signalling molecules crucial to plant survival (Morkunas and Ratajczak, 2014).

On the contrary, plant respiration is usually stimulated after pathogen infection. In particular, the oxidative pentose phosphate pathway (OPP), in which glucose 6-phosphate dehydrogenase (G6PDH) uses glucose 6-phosphate from glycolysis to provide NADPH for NADPH-oxidase-dependent reactive oxygen species (ROS) production (Bolton, 2009). Although ROS are key signalling molecules, a complex antioxidant system, including the ascorbate-glutathione (Asc-GSH) cycle, is needed to maintain plant redox homeostasis and avoid ROS accumulation and cellular damage (Mittler et al., 2004; Foyer and Noctor, 2011).

Pyruvate from glycolysis usually enters the tricarboxylic acid (TCA) cycle to produce energy and reducing power for the electron transport chain. However, under oxidative stress, two steps of this cycle may be inhibited through the γ-aminobutyric acid (GABA) shunt (Bolton, 2009). As an alternative to the TCA cycle, pyruvate decarboxylase (PDC) may convert pyruvate into acetaldehyde and CO_2_ during aerobic fermentation. Acetaldehyde may then enter ethanolic fermentation or the pyruvate dehydrogenase bypass, which diverts toxic fermentative intermediates into acetyl-CoA to re-enter the TCA cycle (Bolton, 2009).

Enhancement of photorespiration, of which glycolate oxidase (GOX) is one of the first enzymes, is also common after pathogen inoculation (Rojas et al., 2014). This may be associated with gas exchange reduction and increased excitation energy after CO_2_ fixation shut-down (Bolton, 2009). Photorespiration is thus induced to dissipate this energy producing ROS, resulting in lipid peroxidation and electrolyte leakage increase (Bolton, 2009). Stomatal closure is related to this process (Bolton, 2009), induced by abscisic acid (ABA) accumulation and overexpression of *SnRK2*, such as *SnRK2.6* (Lim et al., 2015). Together with ABA, salicylic acid (SA), and jasmonic acid (JA) play a key role in plant defence, usually acting as antagonists (Kunkel and Brooks, 2002).

One of the major primary metabolic sinks is the phenylpropanoid pathway. Phenylalanine (Phe) is consumed by phenylalanine ammonia-lyase (PAL) for the synthesis of several compounds crucial for plant survival under stressful scenarios (Pascual et al., 2016). Phenylalanine is synthesised in the shikimate pathway, frequently pathogen-induced (Bolton, 2009). Besides PAL, other important pathogenesis-related (PR) proteins, such as PR3 (chitinase) and PR5 (thaumatin-like protein), are often associated with fungal pathogen resistance (Donoso et al., 2015).

The aim of the present study was to identify physiological changes associated with pathogenesis and plant response in *Pinus* with increasing levels of susceptibility to *F. circinatum* infection (*P. pinea*, *P. pinaster*, and *P. radiata*). Physiological and hormonal analysis and primary metabolite profiles of each species were assessed after *F. circinatum* inoculation, as well as gene expression regulation of target primary and pathogenesis-related genes. The integration of these data will contribute to expand the current knowledge of *Pinus*–*F. circinatum* interaction, which is crucial to define new ways to select resistant phenotypes and control PPC.

## Materials and Methods

### Plant Material

Five-month-old *Pinus radiata* D. Don (Turkish provenance), *Pinus pinaster* Ait. (Portuguese provenance region 2) and *Pinus pinea* L. (Portuguese provenance region 5) seedlings were obtained from Melo & Cancela Lda. (Anadia, Portugal). Plant height (16.36 ± 0.28, 13.67 ± 0.25, and 21.08 ± 0.25 cm, respectively) and stem diameter (0.24 ± 0.03, 0.23 ± 0.03, and 0.35 ± 0.01 cm, respectively) were similar within species. Plants were placed in 200 mL pots with a 3:2 (w/w) peat:perlite mixture and kept in a climate chamber (Fitoclima D1200, Aralab, Sintra, Portugal) under controlled conditions (day/night): 16/8 h photoperiod, 25/20°C temperature, 65/60% relative humidity, and 500 μmol m^2^ s^−1^ photon flux density. Plants were acclimatised for 2 weeks, regularly watered and fertilised weekly (5 mL L^−1^ Frutifol L12, Nufarm, Lisbon, Portugal).

### Fungal Culture

*Fusarium circinatum* FcCa6 isolate was obtained from the Forest Entomology and Pathology Lab at the University of Valladolid (Martínez-Álvarez et al., 2012) and grown on potato dextrose agar (PDA; Scharlau^®^, Barcelona, Spain) at 25°C in the dark until the mycelium covered at least 90% of the Petri dish. Three to five pieces of mycelium (5 mm diameter) were grown under agitation on potato dextrose broth (PDB; VWR Chemicals, Leuven, Belgium) for at least 24 h. Spores were counted using a hemocytometer. Fungus manipulation was performed in biosafety cabinets (Class II). After use, fungal cultures were adequately decontaminated by autoclaving (20 min, 121°C) to ensure safe disposal.

### Plant Inoculation

After acclimatisation, plants of each pine species were assigned to two groups of 15 plants each: non-inoculated controls (C) *vs.* inoculated with *F. circinatum* (F). For inoculation the stem surface was wounded using a sterile scalpel prior to the application of 10^4^ spores. Wounds were sealed with Parafilm^®^. Control group was equally wounded and received an equal volume of sterile distilled water. Controls and inoculated plants were kept in separate climate chambers under the previously described controlled conditions and watering and fertilisation were maintained.

### Evaluation of Visual Symptoms and Sample Collection

The typical disease symptoms of tip dieback, needle wilting and browning, and resin formation were evaluated daily. Sampling was carried out individually for each species when at least 50% of the inoculated plants showed symptoms (Cerqueira et al., 2017). This resulted in three sampling points that occurred 10, 17, and 64 days post-inoculation (d.p.i.) for *P. radiata*, *P. pinaster*, and *P. pinea*, respectively. At each sampling point physiological measurements were performed on symptomatic plants and controls of the corresponding pine species and plant material was harvested for further analysis. Physiological measurements comprised needle gas-exchange related parameters, relative water content (RWC), and electrolyte leakage, and water potential. Plant material was collected, frozen in liquid nitrogen, and kept at −80°C for biochemical and molecular analysis comprising pigment and hormone concentration, gene expression and primary metabolite quantification. To confirm Koch’s postulate, stem cuttings were plated onto PDA and incubated at 25°C for 7 days.

Fungal-infected plant material and experimental material that contacted with the fungus (including soil and containers) were adequately decontaminated by autoclaving.

### Stem Relative Internal Necrosis Length

Relative internal stem lesion length was determined in 5–7 cm longitudinal stem cuts of six biological replicates per treatment. This was calculated as a proportion of the total stem length.

Images of the necrosis were obtained using a zoom stereomicroscope (SMZ1500, Nikon Instruments Europe B.V., Amstelveen, Netherlands) coupled to a high-resolution digital microscope camera (DS-Ri1, Nikon Instruments Europe B.V.) and its controller (DS-U3, Nikon Instruments Europe B.V.). The NIS-Elements Documentation imaging software (v. 64bit 3.22.15, Nikon Instruments Europe B.V.) was used for image acquisition.

### Plant Water Relations

Midday shoot water potential (Ψ_md_, MPa) of six biological replicates per treatment was measured using a Scholander-type pressure chamber (PMS Instrument Co., Albany, OR, United States).

Relative water content was measured according to Escandón et al. (2016). From each of the six replicates per treatment, five needles were collected, fresh weight (FW) was recorded, and needles were transferred to tubes with distilled water. After overnight incubation in the dark at 4°C, excess water was removed from needle surfaces, and turgid weight (TW) was recorded. Needles were oven-dried at 60°C for 1 week and dry weight (DW) was recorded. RWC was calculated as *RWC* (%) = $\frac{\left(\mathrm{FW}-\mathrm{DW}\right)}{\left(\mathrm{TW}-\mathrm{DW}\right)}$ × 100.

### Needle Gas Exchange-Related Parameters

Net CO_2_ assimilation rate (*A*, μmol CO_2_ m^−2^ s^−1^), stomatal conductance (*gs*, mol H_2_O m^−2^ s^−1^), transpiration rate (*E*, mmol H_2_O m^−2^ s^−1^), and sub-stomatal CO_2_ concentration (*Ci*, vpm) were measured using a gas exchange system (LCpro-SD, ADC BioScientific Limited, Hoddesdon, United Kingdom) coupled to a conifer-type chamber. Controlled conditions were maintained inside the chamber: ambient CO_2_ concentration (407.1 ± 1.0 vpm), air flux (200 μmol s^−1^), block temperature (24.5 ± 0.2°C). Light response curves of CO_2_ assimilation (A/PPFD) were performed with the following photosynthetic photon flux density (PPFD): 2,000, 1,500, 1,000, 750, 500, 250, 100, 50 and 0 μmol m^−2^ s^−1^. Measurements were performed at 1,500 μmol m^−2^ s^−1^. Data were recorded when parameters remained stable. Six biological replicates per treatment were measured.

### Electrolyte Leakage (EL)

Three needles from the vegetative growing year per plant were collected into 15 mL Falcon tubes, cut into 1 cm pieces, immersed in 10 mL of Milli-Q water and incubated for 12 h (room temperature, 150 rpm). Conductivity was measured (*C*_exp_), samples were autoclaved (20 min. at 121°C) and cooled at room temperature under agitation for 5 h prior to measurement of maximum conductivity (*C*_m_). Initial Milli-Q water conductivity (*C*_i_) was recorded, as well as after autoclaving (*C*_ii_). Electrolyte leakage was calculated as a measure of cell membrane integrity as *EL*(%) = $\frac{\left(C_{\exp}-C_{i}\right)}{\left(C_{m}-C_{\mathrm{ii}}\right)}$ × 100 (Escandón et al., 2016). Six biological replicates per treatment were considered.

### Chlorophyll and Anthocyanins Concentration

To determine pigment concentration six biological replicates per treatment were considered. Total chlorophylls were quantified according to Sims and Gamon (2002) using 50 mg of frozen pine needles homogenised with acetone/50 mM Tris buffer (80:20, v/v) at pH 7.8. After centrifugation (10,000 × *g*, 5 min, 4°C), supernatants A_663_, A_537_, A_647_, and A_470_ were read. Total chlorophyll concentration was calculated following authors recommendations.

Anthocyanin concentration was determined following extraction in acidified methanol (methanol:HCl, 99:1, v/v) of 50 mg of frozen pine needles (Sanchez-Zabala et al., 2013). The homogenate was immersed in boiling water for 1.5 min and left in the dark at 4°C for 24 h. After centrifugation (10,000 × *g*, 10 min, 4°C), A_530_ and A_657_ were read. Anthocyanin concentration was determined according to the equation proposed by Close et al. (2004), using an extinction coefficient of 33,000 L mol^−1^ cm^−1^.

### Hormones Concentration

Abscisic acid, SA, and JA were extracted and analysed based on Durgbanshi et al. (2005). Needle freeze-dried tissue (50 mg) was extracted in 2 mL of ultrapure water after spiking with 1 ppm [^2^H_6_]-ABA, dehydrojasmonic acid (DHJA), and [^13^C_6_]-SA in a ball mill (MillMix20, Domel, Zelezniki, Slovenija). After centrifugation (4,000 × *g*, 10 min, 4°C), supernatants were recovered and pH adjusted to 3 with 80% acetic acid. The acidified water extract was partitioned twice against 1.5 mL of diethyl ether. The organic upper layer was recovered and vacuum evaporated in a centrifugal concentrator (Speed Vac, Jouan, Saint Herblain Cedex, France). The dry residue was resuspended in 10% MeOH by gentle sonication. The resulting solution was passed through 0.22 μm regenerated cellulose membrane syringe filters (Albet S.A., Barcelona, Spain) and directly injected into a UPLC system (Acquity SDS, Waters Corp., Milford, MA, United States). Analytes were separated by reversed-phase (Nucleodur C18, 1.8 μm 50 × 2.0 mm, Macherey-Nagel, Barcelona, Spain) using a linear gradient of ultrapure H_2_O (A) and MeOH (B) (both supplemented with 0.01% acetic acid) at a 300 μL min^−1^ flow rate. The gradient used was: (0–2 min) 90:10 (A:B), (2–6 min) 10:90 (A:B), and (6–7 min) 90:10 (A:B). Hormones were quantified with a Quattro LC triple quadrupole mass spectrometer (Micromass, Manchester, United Kingdom) connected online to the output of the column through an orthogonal Z-spray electrospray ion source. Quantitation of hormones was achieved using a standard curve. Six biological replicates per treatment were analysed.

### Gene Expression Analysis

RNA was extracted from 50 mg of pine needles of six biological replicates per treatment using the method described by Valledor et al. (2014). cDNA was synthesised using RevertAid Reverse Transcriptase (Thermo Fisher Scientific, Waltham, MA, United States), random hexamers, and 1.5 μg of RNA in 20 μl reactions following the manufacturers specifications. For each treatment, two cDNA pools of three biological replicates each were used.

The relative abundance of transcripts of a set of target genes related to plant primary metabolism and pathogen response was assessed by Real-Time qPCR (Table 1). *Pinus* CDS sequences for each gene were searched for in the NCBI GenBank database^1^ (Clark et al., 2016). For inconclusive search results, a BLASTN search (Altschul et al., 1990) based on known *Arabidopsis* orthologs was employed. Sequences were considered homologous for e-values lower than 10^−10^. Primer pairs were designed using Primer3 v. 0.4.0 (Koressaar and Remm, 2007; Untergasser et al., 2012) under the Geneious Pro 4.8.2^2^ environment (Kearse et al., 2012) assuring that only one gene model of the database was amplified (*in silico* PCR). Analyses were performed using the CFX96 Touch Real-Time PCR detection system (Bio-Rad, Hercules, CA, United States). The 20 μL individual reactions contained 1x Maxima SYBR Green qPCR Master Mix (Thermo Scientific), 0.3 μM of each primer, and 1 μL of 1:10 cDNA. The amplification protocol consisted of 1 × (95°C, 10 min), 45 × (95°C, 15 s; 60°C, 60 s; fluorescence reading) and a final melting curve to assess the quality of amplification (only one gene product per primer pair). Three analytical replicates of the two cDNA pools per treatment and pine species were performed. *Actin* (*ACT*), *Tubulin* (*TUB*), *Hexokinase* (*HXK*), and *Ubiquitin* (*UBQ*) stability under the treatments applied was tested for each pine species using geNorm (Vandesompele et al., 2002). *TUB* and *HXK* were selected as reference genes for *P. radiata*, *ACT* and *TUB* for *P. pinaster*, and *HXK* and *UBQ* for *P. pinea*. The corresponding reference genes were included in every plate. Gene expression results were obtained following the recommendations of Hellemans et al. (2007). Amplicon band size was confirmed by agarose gel for every gene.

**Table 1:** Primer pairs (F: forward; R: reverse) used for real-time qPCR.

Name	*Pinus* accession	*Arabidopsis* accession	e-value	Primer sequence	Functions/putative functions
Actin (*ACT*)	GQ339779.1 (*P. sylvestris*)	–	–	F: TGGACCTTGCTGGGCGTGATCT R: ACAATCTCGCGCTCTGCGGT	Major component of cytoskeleton microfilaments.
β-Tubulin (*TUB*)	KM496536.1 (*P. massoniana*)	–	–	F: AAGGGGGTCAGTGTGGCAACCA R: ACAGCCCGCGGAACAAACCT	Major component of cytoskeleton microtubules.
Hexokinase (*HXK*)	CT580360.1 (*P. pinaster*)	U28214.1	e^−77^	F: TGGCAAGGATGTGGTGGTAGCC R: TCCTCCAGCCAATGTCCCCACT	Responsible for hexose phosphorylation.
Ubiquitin (*UBQ*)	AF461687.1 (*P. pinaster*) (Sanchez et al., 2003)	–	–	F: AGCCCTTATGCCGGAGGGGTTT R: AGTGCGGGACTCCACTGTTCCT	Involved in protein recognition by the proteasome.
Ribulose 1,5-biphosphate carboxylase/oxygenase small subunit (*RuBisCO*)	X13408.1 (*P. thunbergii*) (Yamamoto et al., 1988)	NM_105379.4	e^−36^	F: AACCGTGGTGTCGGCGTTCA R: ACCTGCATGCATCGCACTCG	Carbon fixation into the Calvin cycle. Also involved in photorespiration.
Cell wall invertase (*cwINV*)	AL750756.1 (*P. pinaster*)	NM_112232.4	e^−19^	F: TGGAGAAGGGGGAAAAGCGTGC R: GCAGTGACAGTGGAAGTGCCGT	Cleaves sucrose inducing source-to-sink tissue transformation.
(Cytosolic) Glucose-6-phosphate dehydrogenase (*G6PDH*)	CO171721.1 (*P. taeda*)	AJ010970.1	e^−114^	F: AGGAACCCCATCCCAGCTGTTCA R: TCAGCCTGAGCACATTCGGG	Responsible for the first step of the oxidative pentose phosphate pathway.
Pyruvate decarboxylase (*PDC*)	JQ264496.1 (*P. lambertiana*)	NM_124878.3	e^−11^	F: CCCGCAAACAATGACGTGGGGT R: TGCGAGCAGATGGTCCAGCA	Involved in aerobic fermentation.
Glycolate oxidase (*GOX*)	FN824807.1 (*P. pinaster*)	AY136402.1	e^−55^	F: TGCCGGAGGTGCTGAGGATGAA R: AAAACTCGGGGGCGCAACCT	Responsible for the first step of photorespiration.
Sucrose non-fermenting 1-related protein kinase 2.6 (*SnRK2.6*)	DQ370129.1 (*P. monticola*) (Willyard et al., 2007)	–	–	F: GGTTCATCCATGGACCTGCCAA R: TTGTCGCGCATCAACCTGGC	Involved in the signalling of ABA-induced stomata closure, specially under drought stress.
Phenylalanine ammonia lyase (*pal*)	AY641535.1 (*P. pinaster*)	AY303128.1	e^−146^	F: TGCTGGCCACTGTGAAGCAGA R: TCGCAGAAACGGCCTGGCAA	Involved in the synthesis of molecules crucial for plant survival under stressful scenarios, such as phenylpropanoids, flavonoids, anthocyanins, lignin, lignans, condensed tannins, and salicylic acid.
Thaumatin-like protein (*pr5*)	JQ015859.1 (*P. radiata*)	NM_106161.3	e^−17^	F: AGGAGCGCGTGTGATGCGTT R: TGAAAGTGCTGGTGGCGTCGT	Involved in cell wall damage and formation of pores on the plasma membrane.
Chitinase (*pr3*)	HM219849.1 (*P. contorta*)	NM_112085.4	e^−70^	F: TGGCAACACGGACGCCCATT R: ACCGGCGTCGTTTCTGTGCTT	Hydrolyzation of chitin.

*Gene names and respective GenBank accessions are indicated. Arabidopsis thaliana query sequences used for BLASTN homology search are also shown. Sequences were considered homologous for e-values lower than 10^−10^.*

### Primary Metabolite Analysis

Primary metabolites were extracted using the methanol/chloroform extraction protocol described by Lisec et al. (2006). Thirty milligrams of finely homogenised freeze-dried pine needle were vortex-mixed with 1,400 μL ice-cold 100% (v/v) methanol spiked with 60 μL of 0.2 mg mL^−1^ ribitol in water (internal standard). Samples were incubated for 15 min at 950 rpm and 70°C. After centrifugation (12,000 × *g*, 10 min, room temperature) the supernatant was transferred to a new tube and vortex-mixed with 750 μL chloroform and 1,500 μL water. Tubes were centrifuged at room temperature, 12,000 × *g* for 15 min. A total of 150 μL of the polar (upper) aqueous/methanol phase were evaporated to dryness using a centrifugal concentrator for a minimum of 3 h at 30°C (Vacufuge Plus, Eppendorf, Hamburg, Germany), and stored at −80°C until further analysis. Primary metabolites were derivatized and 1 μL was analysed using an established GC-TOF-MS protocol (Lisec et al., 2006). Biological variations were controlled by analysing quality control (QC) standards by fatty acid methyl esters internal standard markers and a QC standard solution of 41 pure reference compounds (i.e., the most detected and abundant metabolites) throughout the analysis. The obtained GC-TOF-MS files (.cdf format) for each sample were subsequently evaluated using AMDIS v. 2.71. Primary metabolites were annotated using TagFinder (Luedemann et al., 2008) and a reference library of ambient mass spectra and retention indices from the Golm Metabolome Database^3^ (Kopka et al., 2004; Schauer et al., 2005). The relative abundance of primary metabolite levels was normalised to the internal standard (ribitol) and the dry weight of the samples. Hierarchical clustering analysis was performed in R software (R Core Team, 2016) using the ‘heatmap.2’ function from the ‘gplots’ package (Warnes et al., 2016).

### Physiological, Hormone, and Gene Expression Statistical Analysis

Data are presented as mean ± SE (standard error). Physiological, hormone and gene expression data were analysed using SigmaPlot v. 11.0 (Systat Software). Student’s *t*-test was employed to estimate the significance of the results for each pine species independently. When data did not follow Student’s *t-*test assumptions, the non-parametric Mann–Whitney *U* test was performed. Asterisks indicate significant differences between non-inoculated controls (C) and plants inoculated with *F. circinatum* (F) at *p* ≤ 0.05. Gene expression statistical analysis was performed using ΔCq values.

### Integrated Data Analysis

Sparse partial least squares (sPLS) regression is effective to assess relations between variables, predicting the response of a set of variables based on a matrix of predictors (Lê Cao et al., 2008). Gene expression levels were used as predictor matrix for metabolite and physiological responses. mixOmics R package (Rohart et al., 2017) was used to build the regression models. Models were tuned based on total Q2 (threshold for component consideration was established at 0.0975) and variables were selected for individual Q2 > 0.35. This statistical approach was chosen over others such as PCA (principal component analysis) or DA (discriminant analysis) since it allows to consider the flow of information in a living organism (gene → transcript → protein → metabolite/response) and, in consequence, explain how changes in gene expression may lead to differential responses/susceptibilities in inoculated plants.

## Results

### *Pinus* Species Exhibit Differences in Disease Progression

**FIGURE 1:**
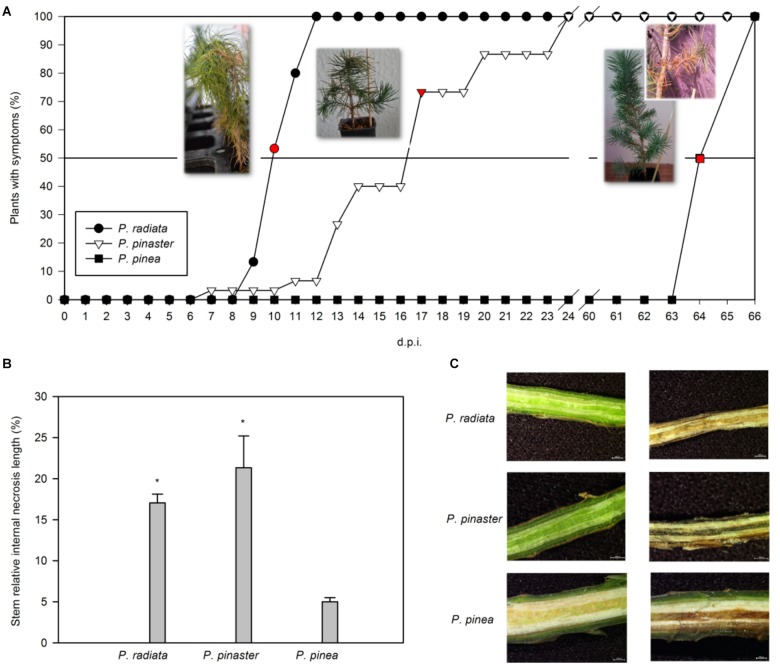
Progression of pitch canker disease in *Pinus*. **(A)** Time course of the percentage of plants inoculated with *F. circinatum* showing symptoms for each pine species. Sampling points were carried out individually for each species when at least 50% of the inoculated plants presented symptoms (red points). Images representative of the symptoms at each sampling point are presented. Days post-infection (d.p.i.). **(B)** Stem relative internal necrosis length of non-inoculated controls (black bars) and of plants inoculated with *F. circinatum* (grey bars) when 50% of the inoculated plants of each species expressed disease symptoms. Data are presented as mean ± SE. Asterisks on grey columns indicate significant differences between treatments for the correspondent species (*p* ≤ 0.05). **(C)** Stem internal necrosis representative of non-inoculated controls (left column) and plants inoculated with *F. circinatum* (right column) of each pine species observed using a zoom stereomicroscope when 50% of the inoculated plants of each species expressed disease symptoms.

About 53% of inoculated *P. radiata* plants presented tip dieback and needle wilting 10 days after *F. circinatum* inoculation (Figure 1A). The same symptoms were registered 8 d.p.i. in ca. 3% of *P. pinaster* plants inoculated with *F. circinatum*, but disease progression was slower, and plants were collected 17 d.p.i. (Figure 1A; ca. 73% of plants with symptoms). *Pinus pinea* remained asymptomatic for longer, revealing slight disease symptoms 64 d.p.i. of branch dieback near the inoculation point and resin formation (Figure 1A). Only *P. radiata* and *P. pinaster* presented significantly higher percentages of relative internal stem necrosis than controls when inoculated with *F. circinatum* (Figure 1B), as represented on the stereomicroscope images (Figure 1C).

Physiological and biochemical experiments were undertaken at 10, 17 and 64 d.p.i for *P. radiata*, *P. pinaster* and *P. pinea* plants, respectively.

### *Fusarium circinatum* Inoculation Is Associated With Changes in Plant Water Relations, Stomatal Closure, and Reduced Photosynthetic Assimilation in Susceptible *Pinus* Species

**FIGURE 2:**
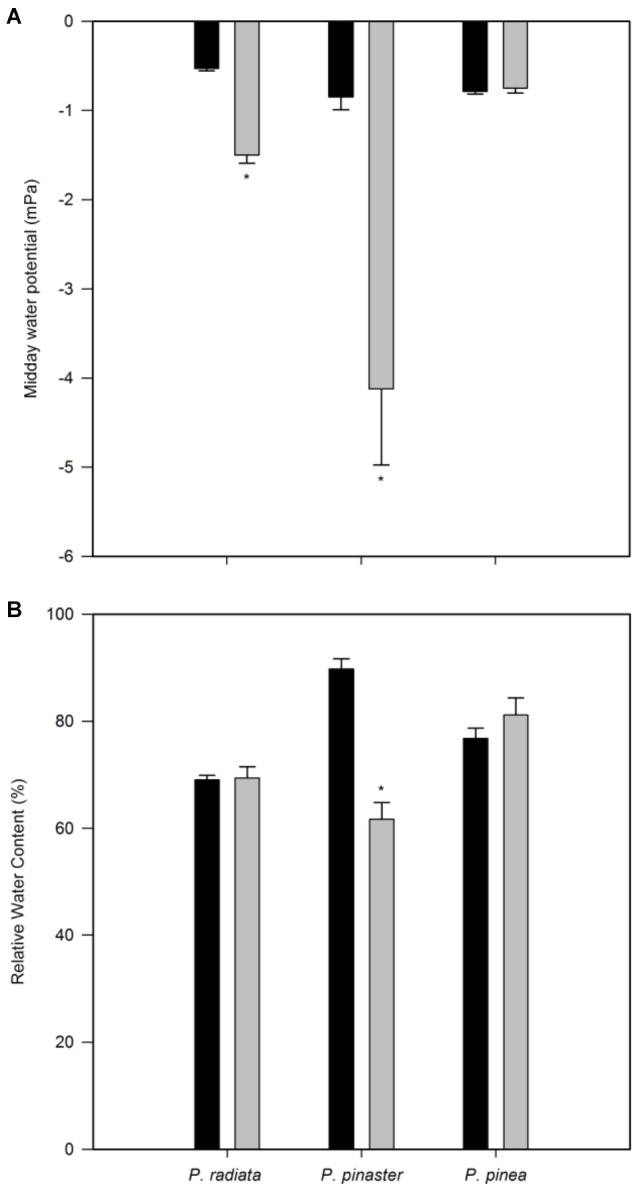
Plant water status of non-inoculated controls (black bars) and plants inoculated with *F. circinatum* (grey bars) when 50% of the inoculated plants of each species expressed disease symptoms. **(A)** Midday water potential. **(B)** Relative water content. Data are presented as mean ± SE. Asterisks on the grey bars indicate significant differences between treatments for the correspondent species (*p* ≤ 0.05).

*Pinus radiata* water potential was significantly decreased after *F. circinatum* inoculation (Figure 2A). While a sharper decrease was observed in *P. pinaster*, no significant changes were observed for *P. pinea*. *Fusarium circinatum* inoculation significantly decreased RWC in *P. pinaster* but not in the other two species (Figure 2B).

**FIGURE 3:**
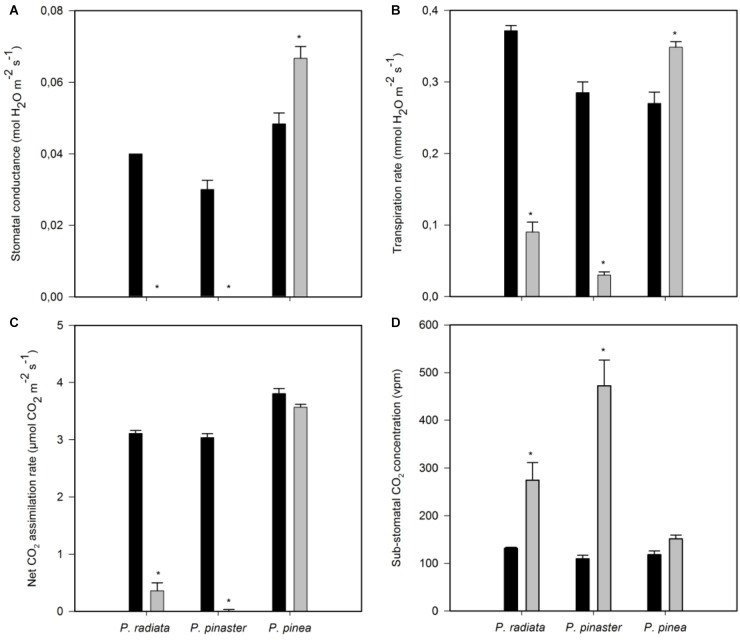
Needle gas exchange-related parameters of non-inoculated controls (black bars) and plants inoculated with *F. circinatum* (grey bars) when 50% of the inoculated plants of each species expressed disease symptoms. **(A)** Stomatal conductance. **(B)** Transpiration rate. **(C)** Net CO_2_ assimilation rate. **(D)** Sub-stomatal CO_2_ concentration. Data are presented as mean ± SE. Asterisks on grey bars indicate significant differences between treatments for the correspondent species (*p* ≤ 0.05).

Inoculation with *F. circinatum* significantly reduced stomatal conductance (*gs*) and transpiration rate (*E*) in *P. radiata* and *P. pinaster* (Figure 3A,B). In contrast, inoculated *P. pinea* showed an increase in *gs* and *E*. After *F. circinatum* inoculation a decrease in net CO_2_ assimilation rate was observed in *P. radiata* and, to a greater extent, in *P. pinaster* but not in *P. pinea* (Figure 3C,D). This was associated with a net increase in needle CO_2_ concentration suggesting that decreased assimilation rates were at least partly caused by biochemical of photochemical limitations.

### *Fusarium circinatum* Inoculation Alters Needle Pigment and Hormones Concentration but Not Electrolyte Leakage

After *F. circinatum* inoculation, total chlorophyll concentration significantly increased in *P. radiata* and *P. pinaster* (Table 2). *Pinus pinea* chlorophyll concentration remained unchanged. Inoculated *P. pinaster* and *P. pinea* showed a significant increase in anthocyanin concentration (Table 2).

**Table 2:** Total chlorophyll and anthocyanin concentration, and electrolyte leakage of non-inoculated control plants (C) and plants inoculated with *F. circinatum* (F) when 50% of the inoculated plants of each species expressed disease symptoms.

Parameter	*P. radiata*		*P. pinaster*		*P. pinea*	
	C	F	C	F	C	F
Total chlorophyll (μmol gFW^−1^)	1.35 ± 0.10	1.85 ± 0.13^∗^	1.80 ± 0.07	2.16 ± 0.08^∗^	1.12 ± 0.03	1.25 ± 0.12
Anthocyanins (mmol gFW^−1^)	0.86 ± 0.11	0.67 ± 0.03	0.80 ± 0.10	1.63 ± 0.08^∗^	0.91 ± 0.14	1.40 ± 0.08^∗^
Electrolyte leakage (%)	7.14 ± 1.39	15.13 ± 4.17	5.82 ± 0.68	18.45 ± 4.72	2.52 ± 0.17	3.03 ± 0.24

*Data are presented as mean ± SE. Asterisks on the F column of each pine species indicate significant differences between C and F for the correspondent species (p ≤ 0.05).*

**FIGURE 4:**
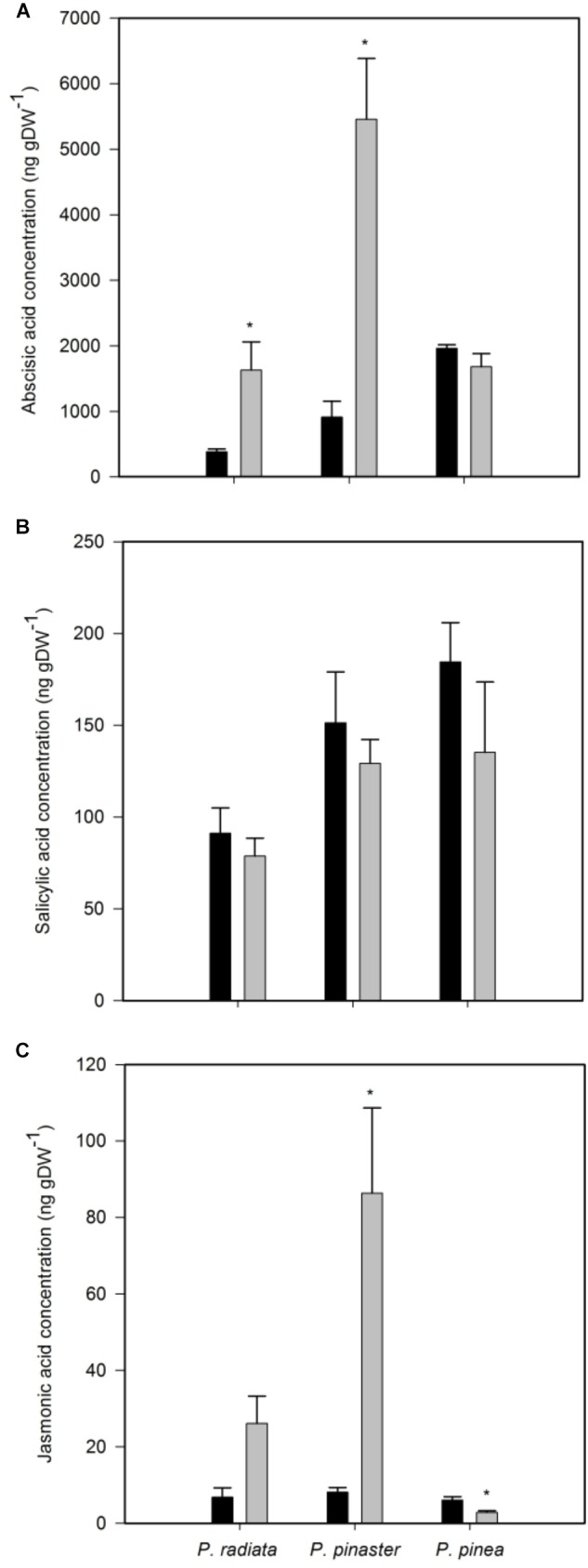
Hormones concentration of non-inoculated controls (black bars) and plants inoculated with *F. circinatum* (grey bars) when 50% of the inoculated plants of each species expressed disease symptoms. **(A)** Abscisic acid. **(B)** Salicylic acid. **(C)** Jasmonic acid. Data are presented as mean ± SE. Asterisks on grey bars indicate significant differences between treatments for the correspondent species (*p* ≤ 0.05).

Inoculation of *P. radiata* and *P. pinaster* led to a significant increase of ABA concentration (Figure 4A). On the other hand, *F. circinatum* inoculation did not induced changes in ABA concentration in *P. pinea* (Figure 4A) or in SA concentration in any pine species (Figure 4B). Inoculated *P. pinaster* significantly increased JA concentration (Figure 4C). In contrast, no change was observed in inoculated *P. radiata* and a slight but significant decrease in JA concentration was observed in inoculated *P. pinea*.

No significant changes were observed after *F. circinatum* inoculation in EL in any *Pinus* species (Table 2).

### Susceptible *Pinus* Species Exhibit Stronger Changes in Gene Expression Than the Tolerant Species

**FIGURE 5:**
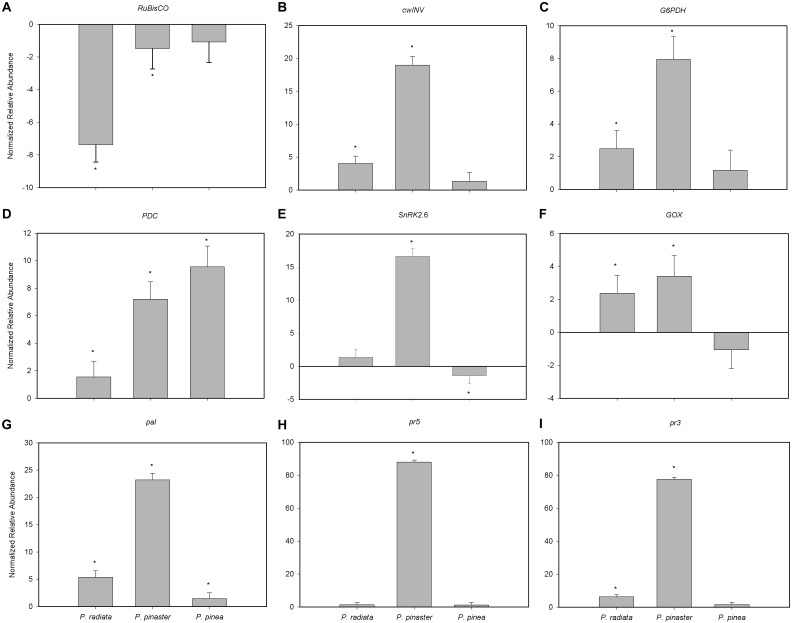
Relative abundance of primary metabolism- and pathogenesis-related genes in *Pinus* inoculated with *F. circinatum* with respect to their non-inoculated controls when 50% of the inoculated plants of each species expressed disease symptoms. **(A)** Ribulose 1,5-biphosphate carboxylase/oxygenase small subunit (*RuBisCO*). **(B)** Cell wall invertase (*cwINV*). **(C)** (Cytosolic) Glucose-6-phosphate dehydrogenase (*G6PDH*). **(D)** Pyruvate decarboxylase (*PDC*). **(E)** Glycolate oxidase (*GOX*). **(F)** Sucrose non-fermenting 1-related protein kinase 2.6 (*SnRK2.6*). **(G)** Phenylalanine ammonia lyase (*pal*). **(H)** Thaumatin-like protein (*pr5*). **(I)** Chitinase (*pr3*). The abundance of transcripts in plants inoculated with *F. circinatum* for each pine species is represented considering its correspondent non-inoculated controls. Data are presented as mean ± SE. Asterisks indicate significant differences between non-inoculated controls and plants inoculated with *F. circinatum* for each species (*p* ≤ 0.05).

*RuBisCO* transcript abundance was significantly reduced in the presence of *F. circinatum* in *P. pinaster* and, to a greater extent, in *P. radiata* (Figure 5A). On the contrary, *F. circinatum* inoculation resulted in a significant increase in abundance of transcripts encoding *cwINV*, *G6PDH*, *PDC*, *GOX*, *pal* and *pr3* in *P. radiata* which were enhanced to an even greater extent in *P. pinaster* (Figure 5B–D,F,G,I, respectively). *PDC* and *pal* transcripts were also significantly increased in inoculated *P. pinea* and while the fold-change in *PDC* transcripts was the highest of the three species in *P. pinea* (Figure 5D) relative to the other species, the change in *pal* transcript abundance after *F. circinatum* inoculation was much lower in *P. pinea* than in the other species (Figure 5G). Transcript abundance of *pr5* was only significantly altered in *P. pinaster* following *F. circinatum* inoculation where transcripts increased by almost 90-fold (Figure 5H). Transcripts encoding *SnRK2.6* were unaffected by *F. circinatum* inoculation of *P. radiata* while this treatment increased transcript abundance in *P. pinaster* and decreased transcript abundance in *P. pinea* (Figure 5E).

### *Fusarium circinatum* Inoculation Significantly Alters Primary Metabolism

**FIGURE 6:**
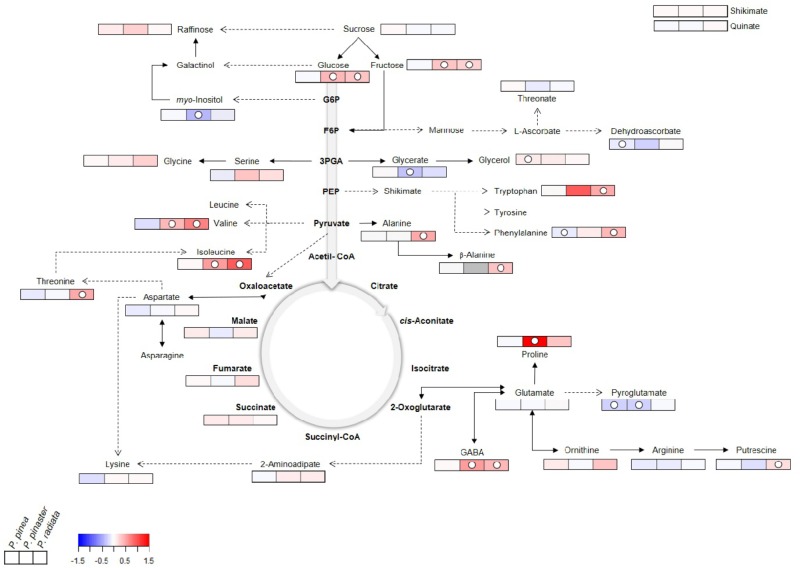
Primary metabolite changes occurring in *Pinus* inoculated with *F. circinatum* with respect to their non-inoculated controls when 50% of the inoculated plants of each species expressed disease symptoms. Relative values are normalised to the internal standard (ribitol) and dry weight (DW) of the samples. Significant changes are indicated as ° *P* < 0.05, with respect to controls for each one of the species represented in the three different squares. False-colour imaging was performed on log_10_-transformed GC-TOF-MS data. Grey-colour square represents not detected (n.d.) value.

GC-TOF-MS profiling allowed the identification of a total of 33 primary metabolites in *Pinus*. These included 18 amino acids and derivatives (AA), six sugars and sugar alcohols (SS), five organic acids (OA), and four other (O) metabolites (Figure 6, Supplementary Figure S1, and Supplementary Table S1). Changes in the relative abundance of primary metabolites from all chemical groups with the exception of OA were observed following inoculation with *F. circinatum*.

Significant increases occurred in several AA in *P. radiata* after *F. circinatum* inoculation: GABA, β-alanine, alanine, Phe, threonine, isoleucine (Ile), valine, tryptophan (Trp), and putrescine. The relative abundance of some of these metabolites was also enhanced by the presence of *F. circinatum* in *P. pinaster*, either to a greater extent (GABA) or at lower levels (Ile and valine) than in *P. radiata*. Proline (Pro) levels were also significantly increased by over 30-fold upon *F. circinatum* infection in *P. pinaster*. In inoculated *P. pinea*, of the AA identified only Phe and pyroglutamate were significantly decreased, and none of the AA were significantly increased.

Glucose (Glc) and Fru levels were significantly increased following pathogen infection in *P. radiata* and *P. pinaster* but remained unchanged in *P. pinea*. Sugar alcohols content was altered by PPC: a significant increase was registered for glycerol in inoculated *P. pinea* and *myo*-inositol levels significantly decreased in inoculated *P. pinaster*.

Upon *F. circinatum* inoculation, significant lower levels of glycerate were found only in *P. pinaster* while pyroglutamate levels were significantly lower in *P. pinea* and *P. pinaster*. Additionally, a significant decrease of dehydroascorbate was detected only in *P. pinea*.

### Integrated Data Analysis Clearly Separates Resistant From Susceptible *Pinus* Species Responses

**FIGURE 7:**
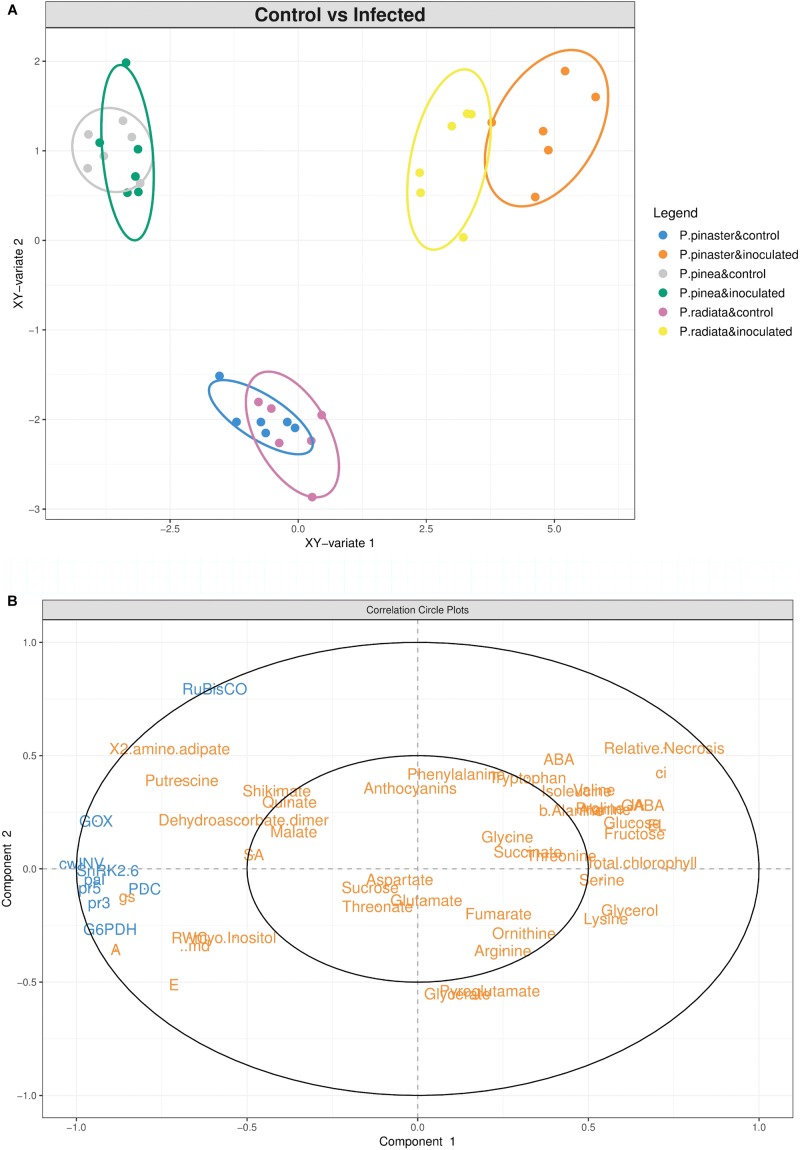
Integrated data analysis. **(A)** Sparse partial least squares (sPLS) regression analysis of the complete dataset of physiological, hormonal, gene expression and primary metabolism alterations occurring in *Pinus* inoculated with *F. circinatum* and their respective non-inoculated controls when 50% of the inoculated plants of each species expressed disease symptoms. First two components are plotted in the graph. **(B)** Correlation circle plot of the components represented in the sPLS regression analysis. Gene expression levels (blue) were used as predictor matrix for metabolite and physiological responses (orange).

The sPLS analysis highlights the different *Pinus* response profiles under *F. circinatum* infection (Figure 7). A clear separation between control and inoculated samples was found for *P. radiata* and *P. pinaster*, both on the vertical (*y*-axis) and horizontal axis (*x*-axis) (Figure 7A). For *P. pinea* no separation of the control and inoculated samples scores occurred. The positioning of inoculated *P. radiata* and *P. pinaster* samples corresponds to a cluster of most of the metabolites on the positive side of the *x*-axis (Figure 7A,B), with *P. pinaster* samples showing higher scores. Together with this, relative necrosis, Ci, ABA and total chlorophyll are placed in the positive side of the *y*-axis (Figure 7B). On the negative part of the *x*-axis we find the genes analysed, water relations, A, E, gs, and SA, influencing the overlap observed in *P. pinea* samples, in particular *RubisCO* and *GOX* expression, and SA on the positive side of the *y*-axis.

## Discussion

The timing and intensity of disease symptoms observed after *F. circinatum* inoculation in the *Pinus* species tested agrees with the levels of susceptibility described by Bragança et al. (2009) and Iturritxa et al. (2013). The overview of our data through sPLS analysis also revealed different profiles of response upon pathogen inoculation: *P. radiata* and, especially *P. pinaster* were observed to be more responsive to pathogen inoculation; while *P. pinea* maintained a phenotype more similar to that of controls, in accordance with its tolerance to PPC.

### *Fusarium circinatum* Inoculation Affects Plant Water Status and Photosynthesis and Induces Sink Metabolism in Susceptible *Pinus* Species

Martín-Rodrigues et al. (2013) stated that *F. circinatum* enters *P. radiata* stem blocking water and nutrient flux, which is supported by the Ψ_md_ decrease reported here in the susceptible *Pinus* species and previously in *P. radiata* (Cerqueira et al., 2017). Under this water deprivation-like scenario *P. radiata* and *P. pinaster* closed their stomata to reduce water loss by transpiration. In *Arabidopsis* this process is regulated by SnRK2, such as SnRK2.6, whose activity is promoted by ABA accumulation (Lim et al., 2015). The accumulation of ABA upon *F. circinatum* has been previously reported in *P. radiata* (Cerqueira et al., 2017), and was found here also for *P. pinaster*.

In accordance with a greater water limiting-like status, only inoculated *P. pinaster* presented an up-regulation of *SnRK2.6* and *pr5* together with Pro accumulation. Besides conferring tolerance to soybean cyst nematode (Matthews et al., 2014), overexpression of *pr5* was also observed in drought-tolerant *Arabidopsis* (Liu et al., 2013); while Pro accumulation is widely associated with plant stress tolerance and a well-known osmolyte (Verbruggen and Hermans, 2008). The enhanced resilience of *P. pinaster* to water-limiting conditions in comparison with *P. radiata* has been previously studied (Smith et al., 2014) and could in part explain the earlier symptom development in *P. radiata* at lower thresholds of water deficit.

The increase of chlorophyll concentration in the susceptible *Pinus* species is at odds with previous studies indicating that photosynthetic gene expression including those associated with pigment biosynthesis is downregulated in a range of species upon pathogen attack (Bilgin et al., 2010). In fact, net CO_2_ assimilation rate was impaired in inoculated *P. radiata*, as reported in Cerqueira et al. (2017), and in *P. pinaster*. Although this is in part explained by reduced stomatal conductance, the accumulation of intercellular CO_2_ suggests that also biochemical limitations impaired photosynthesis in these species, as shown by the down-regulation of *RuBisCO* after *F. circinatum* inoculation. The down-regulation of photosynthetic genes under pathogen attack is known to be regulated by source-to-sink tissue transformation through cleavage of sucrose into Fru and Glc by *cwINV* (Berger et al., 2007; Bolton, 2009), as verified by the overexpression of *cwINV* and accumulation of Glc and Fru in both species.

### *Pinus*–*F. circinatum* Interaction Leads to Amino Acid Accumulation and Overexpression of Pathogenesis-Related Genes in Susceptible *Pinus* Species

The overexpression of *G6PDH* in inoculated *P. radiata* and *P. pinaster* suggests the activation of the OPP under *F. circinatum* infection. Compounds from the OPP are involved in the biosynthesis of amino acids (AA), such as Trp (Maeda and Dudareva, 2012), which accumulated upon pathogen inoculation in the susceptible *Pinus* species. This may be a means to induce the production of secondary metabolites involved in defence response, as reported in rice infected by *Bipolaris oryzae* (Ishihara et al., 2008). However, besides being a common defence response against pathogens, the general accumulation of other AA in *P. radiata* may also reflect *F. circinatum* manipulation of plants metabolism to enhance nitrogen availability in its favour (Fagard et al., 2014). In both susceptible species also the GABA shunt may be stimulated by *F. circinatum* to use it as a nutrient source, as occurred with *Cladosporium fulvum* in tomato (Solomon and Oliver, 2002). The decline in *P. pinaster myo*-inositol levels also indicates that it may be being consumed by the pathogen as inositols are essential for fungal growth (reviewed by Valluru and Van den Ende, 2011).

Phenylalanine plays a key role in linking plant primary and secondary metabolism. It is used by *pal* for the synthesis of several compounds crucial to determine plant survival under stressful scenarios (Pascual et al., 2016). In inoculated *P. pinaster* and *P. pinea* the available Phe could be being used for anthocyanin biosynthesis, important antioxidants that may confer early tolerance to *F. circinatum* in these species. The regulation of *pal* expression by the sucrose/hexose ratio, as the impairment of *pal* activity in mutant tobaccos silenced for *cwINV* indicated (Essmann et al., 2008), was verified for every pine species upon *F. circinatum* inoculation.

Although *pal* up-regulation has been associated with *F. circinatum* resistance in *P. radiata* genotypes (Donoso et al., 2015) and in *P. patula* seedlings treated with chitosan (Fitza et al., 2011, 2013), Morse et al. (2004) suggested that PR genes are induced only in susceptible *Pinus* spp. during PPC development. In fact, the unchanged expression of *pr3* and *pr5* in *P. pinea* is also in accordance with this hypothesis. The overexpression of *pr3* in *P. radiata* and *P. pinaster* further supports that plant chitinases, often associated with fungi resistance, are not involved in PPC tolerance, as observed by Davis et al. (2002) in *P. taeda* and by Donoso et al. (2015) in *P. radiata*.

### *P. pinea* Presented Key Responses Contrasting With the Most Susceptible Species in the Presence of *F. circinatum*

The decrease of dehydroascorbate (DHA) in inoculated *P. pinea* suggests changes in the ascorbate-glutathione (Asc-GSH) cycle, a key component of stress response given Asc antioxidant functions against H_2_O_2_. Vanacker et al. (1998) found a decrease of apoplastic DHA and increased DHA reductase (DHAR) activity in *Blumeria graminis* resistant barley. Moreover, Chen and Gallie (2004) demonstrated that DHAR overexpression in guard cells resulted in an increased Asc redox state, reducing H_2_O_2_ levels and promoting stomata opening and transpiration. These studies are in line with the maintenance of ABA levels, down-regulation of *SnRK2.6*, stomata opening and increased transpiration observed in inoculated *P. pinea*. However, further studies on the regulation of the Asc-GSH cycle in *Pinus* under *F. circinatum* infection are needed. Glycerol accumulation in *P. pinea* may also contribute to enhanced tolerance to *F. circinatum*, as reported in several abiotic stresses, including oxidative stress (Eastmond, 2004), and in wheat powdery mildew (Li et al., 2016).

The overexpression of *PDC* in all *Pinus* species suggests the deviation of pyruvate from the TCA cycle in a *F. circinatum* infection scenario. This response was intensified according to the increasing level of species tolerance, which may indicate species-specific metabolic shifts involved in conferring tolerance to *F. circinatum* in *Pinus*. In accordance, Tadege et al. (1999) proved that tobacco mutants overexpressing *PDC* resisted to *Phytophthora infestans*. PDC converts pyruvate into acetaldehyde, which can enter ethanolic fermentation or the pyruvate dehydrogenase bypass (Bolton, 2009). The later was involved in *Lr34*-mediated wheat resistance to *Puccinia triticina* but it was not maintained in later stages of infection, explaining the partial resistance observed (Bolton et al., 2008).

The up-regulation of *GOX* in inoculated *P. radiata* and *P. pinaster* contrasts with the trend down-regulation registered in *P. pinea*, which highly influences the distribution of the latter in the sPLS plot. However, *RuBisCO*, responsible for an oxygenase reaction in photorespiration (Rojas et al., 2014), was down-regulated after *F. circinatum* infection and the levels of the photorespiration intermediate glycerate declined in the susceptible *Pinus* species. Therefore, it is more likely that *GOX* regulation is related to the lower induction of *PDC*, which is conceivably associated with increased needle CO_2_ even when stomata are closed.

Furthermore, although *GOX* is known to activate SA-mediated defence responses by Pro and its derivates, no significant changes were found in SA levels for every species. Rather, an increase in JA concentration was observed for inoculated *P. radiata* and *P. pinaster*, which is known to be involved in plant response against necrotrophic fungi and counteract SA biosynthesis (Kunkel and Brooks, 2002). A contrasting response was found for *P. pinea* with a decrease of JA levels upon *F. circinatum* infection. Interestingly, SA endogenous levels in controls increase according to the tolerance to *F. circinatum* infection as evidenced in the sPLS analysis. A significantly higher basal level of SA was also found in an *Eucalyptus grandis* clone resistant to *Chrysoporthe austroafricana* in comparison with a susceptible clone (Mangwanda et al., 2015). This may also be a potential basal mechanism of resistance against *F. circinatum* infection.

Our work provides new insights into the physiological changes associated with pathogenesis and plant response in the *Pinus*–*F. circinatum* interaction. By using an integrated approach, we were able to explore this pathosystem at different cellular levels and we link molecular changes to physiological traits with a focus on primary plant metabolism. In general *P. radiata* and *P. pinaster* showed similar response profiles, with pathogen inoculation affecting plant water status, photosynthesis and amino acids concentration, and inducing sink metabolism and pathogenesis-related genes expression. For the resistant *P. pinea* specific responses were found upon pathogen inoculation, mainly related with changes in hormone concentration, stomatal opening and transpiration rate increase, glycerol accumulation and greater *PDC* overexpression. These results provide interesting avenues for future research to unveil *Pinus–F. circinatum* interaction. Once we understand this relationship we will have better tools to fight PPC.

## Author Contributions

LV, RDH, AA, and GP designed and supervised the experimental procedure. AG-C designed and supervised hormones quantification. JA, BC, and GP performed the experiments and physiological characterisation. JA performed hormone quantification, gene expression experiments, and analysed the data. CA and AMR performed primary metabolite analysis. LV conducted the multivariate analysis. JA wrote the manuscript. All authors discussed the data and reviewed the manuscript.

## Conflict of Interest Statement

The authors declare that the research was conducted in the absence of any commercial or financial relationships that could be construed as a potential conflict of interest.

## Footnotes

1 https://www.ncbi.nlm.nih.gov/genbank/

2 http://www.geneious.com

3 http://gmd.mpimp-golm.mpg.de/
