# Supplementary material for: Pinus Susceptibility to Pitch Canker Triggers Specific Physiological Responses in Symptomatic Plants: An Integrated Approach
Source: Front Plant Sci. 2019 Apr 24;10:509. doi: 10.3389/fpls.2019.00509 (PMC6491765; doi:10.3389/fpls.2019.00509)
Supplement: Supplementary file 1 [file Image_1.pdf]

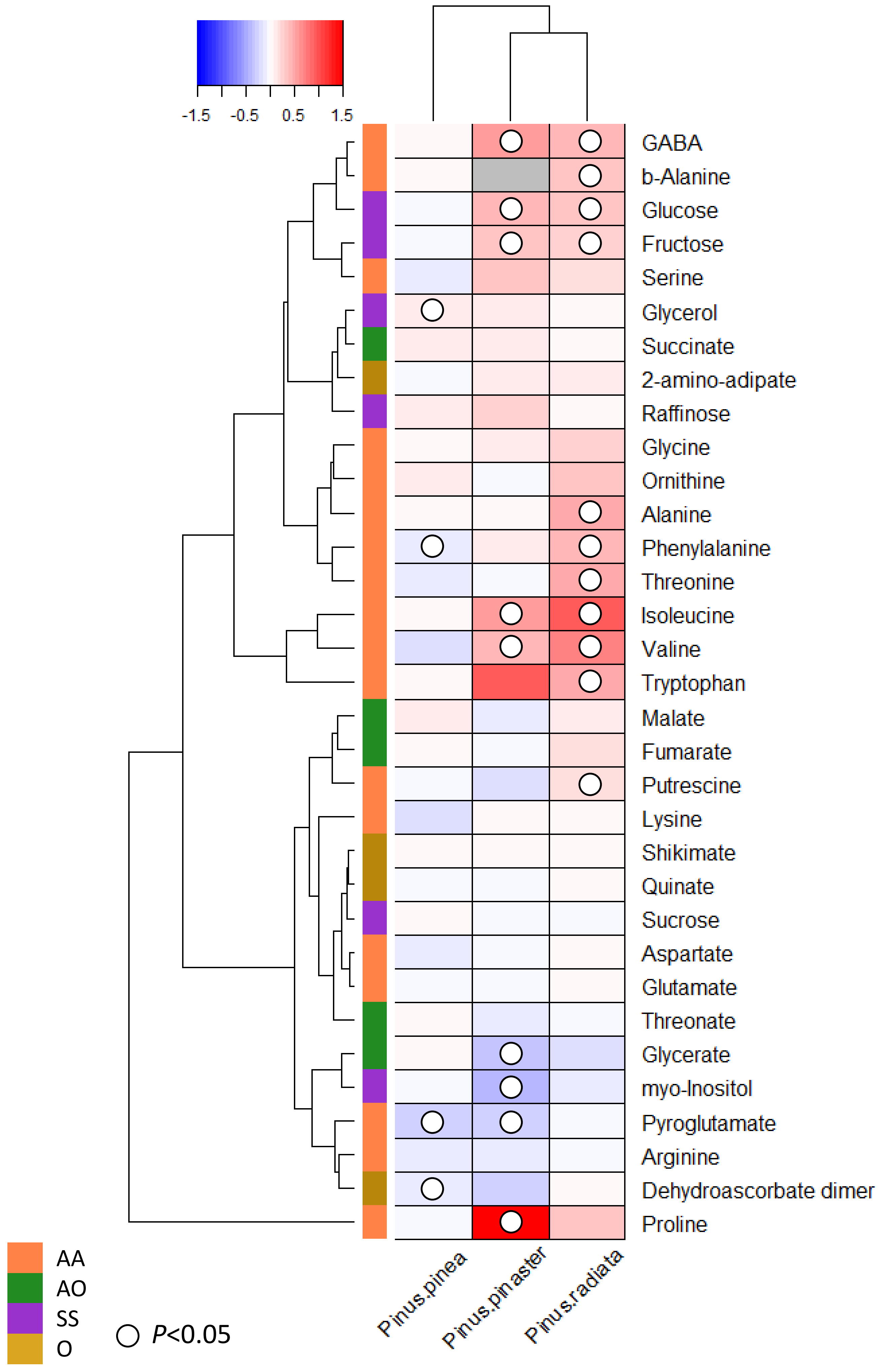

-1.5
-0.5
0.5
1.5
GABA
b-Alanine
Glucose
Fructose
Serine
Glycerol
Succinate
2-amino-adipate
Raffinose
Glycine
Ornithine
Alanine
Phenylalanine
Threonine
Isoleucine
Valine
Tryptophan
Malate
Fumarate
Putrescine
Lysine
Shikimate
Quinate
Sucrose
Aspartate
Glutamate
Threonate
Glycerate
myo-Inositol
Pyroglutamate
Arginine
Dehydroascorbate dimer
Proline
Pinus.pinea
Pinus.pinaster
Pinus.radiata
AA
AO
SS
O
P<0.05

Figure S1 | Heatmap of primary metabolite changes occurring in *Pinus* inoculated with *F. circinatum* with respect to their non-inoculated controls when 50% of the inoculated plants of each species expressed disease symptoms. Relative values are normalized to the internal standard (ribitol) and dry weight (DW) of the samples. Values are presented as means ± SE of six independent measurements. Significant changes using Student's *t* test are indicated as ○ $P < 0.05$, with respect to controls. False-colour imaging was performed on $\log_{10}$-transformed GC-TOF-MS data. Metabolites are grouped in sugars & sugar alcohols (SS), amino acids & derivatives (AA), organic acids (OA) and others (O).
