# Supplementary material for: Pinus Susceptibility to Pitch Canker Triggers Specific Physiological Responses in Symptomatic Plants: An Integrated Approach
Source: Front Plant Sci. 2019 Apr 24;10:509. doi: 10.3389/fpls.2019.00509 (PMC6491765; doi:10.3389/fpls.2019.00509)
Supplement: Supplementary file 2 [file Table_1.pdf]

Table S1 | Relative abundance of primary metabolites in *Pinus* inoculated with *F. circinatum* with respect to their non-inoculated controls when 50% of the inoculated plants of each species expressed disease symptoms. Relative values are normalized to the internal standard (ribitol) and dry weight (DW) of the samples. Data are presented as mean ± SE of six independent measurements. Values in bold-type indicate significant differences calculated using the Student's t test ($p < 0.05$) with respect to the control. Metabolites are grouped in sugars & sugar alcohols (SS), amino acids & derivatives (AA), organic acids (OA) and others (O). n.d. - not detected.

Class	Metabolite	*Pinus pinea*	*Pinus pinaster*	*Pinus radiata*
AA	Alanine	1.16 ± 0.07	1.12 ± 0.17	**2.83 ± 0.26**
AA	b-Alanine	1.12 ± 0.12	n.d.	**2.37 ± 0.29**
AA	Arginine	0.77 ± 0.18	0.71 ± 0.17	0.96 ± 0.37
AA	Aspartate	0.84 ± 0.10	0.95 ± 0.20	1.1 ± 0.04
AA	GABA	1.05 ± 0.14	**3.41 ± 0.36**	**2.6 ± 0.43**
AA	Glutamate	0.86 ± 0.09	0.87 ± 0.13	1.09 ± 0.03
AA	Glycine	1.06 ± 0.08	1.27 ± 0.28	1.84 ± 0.39
AA	Isoleucine	1.03 ± 0.51	**3.75 ± 0.55**	**8.72 ± 2.09**
AA	Lysine	0.60 ± 0.11	1.06 ± 0.34	1.15 ± 0.42
AA	Ornithine	1.23 ± 0.36	0.88 ± 0.14	2.00 ± 0.58
AA	Phenylalanine	**0.75 ± 0.07**	1.41 ± 0.31	**2.6 ± 0.68**
AA	Proline	0.87 ± 0.14	**31.65 ± 3.42**	2.04 ± 0.50
AA	Putrescine	0.97 ± 0.06	0.63 ± 0.20	**1.63 ± 0.20**
AA	Pyroglutamate	**0.56 ± 0.05**	**0.57 ± 0.07**	0.94 ± 0.17
AA	Serine	0.78 ± 0.17	2.06 ± 0.36	1.51 ± 0.25
AA	Threonine	0.75 ± 0.06	0.94 ± 0.22	**2.89 ± 0.62**
AA	Tryptophan	1.07 ± 0.44	8.81 ± 4.27	**3.27 ± 0.98**
AA	Valine	0.67 ± 0.09	**2.81 ± 0.37**	**5.35 ± 1.45**
OA	Succinate	1.2 ± 0.16	1.25 ± 0.28	1.17 ± 0.18
OA	Fumarate	1.03 ± 0.11	0.85 ± 0.19	1.48 ± 0.21
OA	Malate	1.34 ± 0.27	0.76 ± 0.11	1.32 ± 0.18
OA	Glycerate	1.18 ± 0.19	**0.46 ± 0.08**	0.71 ± 0.04
OA	Threonate	1.07 ± 0.15	0.82 ± 0.08	0.84 ± 0.08
SS	Fructose	0.91 ± 0.07	**2.26 ± 0.46**	**1.76 ± 0.17**
SS	Glucose	0.98 ± 0.13	**2.48 ± 0.50**	**2.21 ± 0.28**
SS	Glycerol	**1.27 ± 0.04**	1.37 ± 0.12	1.02 ± 0.06
SS	myo-Inositol	0.96 ± 0.11	**0.41 ± 0.09**	0.74 ± 0.06
SS	Raffinose	1.21 ± 0.04	1.85 ± 0.45	1.03 ± 0.13
SS	Sucrose	1.03 ± 0.04	0.96 ± 0.06	0.99 ± 0.05
O	2-Amino-adipate	0.97 ± 0.06	1.36 ± 0.23	1.24 ± 0.14
O	Dehydroascorbate	**0.75 ± 0.06**	0.53 ± 0.13	1.04 ± 0.18
O	Quinate	0.95 ± 0.02	0.97 ± 0.14	1.12 ± 0.05
O	Shikimate	1.03 ± 0.06	1.02 ± 0.06	1.07 ± 0.04

AA
OA
SS
O
